# Supplementary material for: A Critical View of the Application of the APEX Software (Aqueous Photochemistry of Environmentally-Occurring Xenobiotics) to Predict Photoreaction Kinetics in Surface Freshwaters
Source: Molecules. 2019 Dec 18;25(1):9. doi: 10.3390/molecules25010009 (PMC7017383; doi:10.3390/molecules25010009)
Supplement: Supplementary file 1 [file molecules-25-00009-s001.zip › 3.2.4_gcc-4.4.0.zip!/3.2.4_gcc-4.4.0/3.2.4_gcc-4.4.0/doc/octave/html/interpreter/Single-Precision-Data-Types.html]

Single Precision Data Types - Untitled


Next: Integer Data Types,
Previous: Ranges,
Up: Numeric Data Types


---

### 4.3 Single Precision Data Types

Octave includes support for single precision data types, and most of the
functions in Octave accept single precision values and return single
precision answers. A single precision variable is created with the
`single` function.

— Built-in Function: **single** (x)  
> Convert x to single precision type.
>
>
>
> **See also:** double.

for example

```
     sngl = single (rand (2, 2))
          => sngl =
             0.37569   0.92982
             0.11962   0.50876
     class (sngl)
         => single
```

Many functions can also return single precision values directly. For
example

```
     ones (2, 2, "single")
     zeros (2, 2, "single")
     eye (2, 2,  "single")
     rand (2, 2, "single")
     NaN (2, 2, "single")
     NA (2, 2, "single")
     Inf (2, 2, "single")
```

will all return single precision matrices.
